# 2-[(*E*)-2-(4-Ethoxy­phen­yl)ethen­yl]-1-methyl­quinolinium iodide dihydrate

**DOI:** 10.1107/S1600536810010548

**Published:** 2010-03-27

**Authors:** Hoong-Kun Fun, Kullapa Chanawanno, Thawanrat Kobkeatthawin, Suchada Chantrapromma

**Affiliations:** a X-ray Crystallography Unit, School of Physics, Universiti Sains Malaysia, 11800 USM, Penang, Malaysia; b Crystal Materials Research Unit, Department of Chemistry, Faculty of Science, Prince of Songkla University, Hat-Yai, Songkhla 90112, Thailand

## Abstract

In the title compound, C_20_H_20_NO^+^·I^−^·2H_2_O, the cation is almost planar (r.m.s. deviation = 0.038 Å) and exists in an *E* configuration. The dihedral angle between the quinolinium ring system and the benzene ring is 0.7 (4)°. In the crystal structure, the cations are stacked in an anti-parallel manner along [100] with π–π inter­actions between the pyridinium and ethoxy­benzene rings [centroid–centroid distance = 3.678 (5) Å]. The cations, iodide anions and water mol­ecules are linked together through O—H⋯O, O—H⋯I and C—H⋯I hydrogen bonds into a two-dimensional network parallel to (001).

## Related literature

For background to non-linear optical materials research, see: Kagawa *et al.* (1994 ▶); Williams (1984 ▶). For the anti­bacterial activity of quinoline derivatives, see: Hopkins *et al.* (2005 ▶); Kaminsky & Meltzer (1968 ▶); Musiol *et al.* (2006 ▶); O’Donnell *et al.* (2010 ▶). For a related structure, see: Laksana *et al.* (2008 ▶). For bond-length data, see: Allen *et al.* (1987 ▶). For the stability of the temperature controller used in the data collection, see: Cosier & Glazer (1986 ▶).
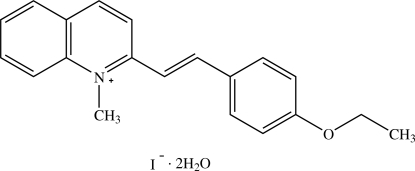


## Experimental

### 

#### Crystal data

C_20_H_20_NO^+^·I^−^·2H_2_O
*M* *_r_* = 453.30
Triclinic, 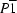

*a* = 8.2450 (9) Å
*b* = 10.6676 (12) Å
*c* = 12.2492 (14) Å
α = 85.789 (2)°
β = 70.516 (2)°
γ = 71.272 (2)°
*V* = 961.21 (19) Å^3^
*Z* = 2
Mo *K*α radiation
μ = 1.68 mm^−1^
*T* = 100 K
0.49 × 0.08 × 0.05 mm

#### Data collection

Bruker APEX DUO CCD area-detector diffractometer
Absorption correction: multi-scan (*SADABS*; Bruker, 2009 ▶) *T*_min_ = 0.493, *T*_max_ = 0.927
11172 measured reflections
3910 independent reflections
3551 reflections with *I* > 2σ(*I*)
*R*_int_ = 0.033

#### Refinement

*R*[*F* ^2^ > 2σ(*F* ^2^)] = 0.074
*wR*(*F* ^2^) = 0.211
*S* = 1.15
3910 reflections
220 parameters
20 restraints
H-atom parameters constrained
Δρ_max_ = 2.43 e Å^−3^
Δρ_min_ = −0.85 e Å^−3^

### 

Data collection: *APEX2* (Bruker, 2009 ▶); cell refinement: *SAINT* (Bruker, 2009 ▶); data reduction: *SAINT*; program(s) used to solve structure: *SHELXTL* (Sheldrick, 2008 ▶); program(s) used to refine structure: *SHELXTL*; molecular graphics: *SHELXTL*; software used to prepare material for publication: *SHELXTL* and *PLATON* (Spek, 2009 ▶).

## Supplementary Material

Crystal structure: contains datablocks global, I. DOI: 10.1107/S1600536810010548/ci5058sup1.cif

Structure factors: contains datablocks I. DOI: 10.1107/S1600536810010548/ci5058Isup2.hkl

Additional supplementary materials:  crystallographic information; 3D view; checkCIF report

## Figures and Tables

**Table 1:** Hydrogen-bond geometry (Å, °)

*D*—H⋯*A*	*D*—H	H⋯*A*	*D*⋯*A*	*D*—H⋯*A*
O2*W*—H1*W*2⋯O1*W*	0.83	1.99	2.711 (11)	143
O1*W*—H2*W*1⋯I1^i^	0.84	2.77	3.588 (7)	163
O2*W*—H2*W*2⋯I1^ii^	0.84	2.87	3.579 (7)	144
C2—H2*A*⋯I1^iii^	0.93	3.02	3.814 (10)	145
C7—H7*A*⋯I1^i^	0.93	2.89	3.708 (10)	148

Symmetry codes: (i) 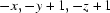; (ii) 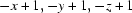; (iii) 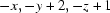.

**Fig. 1.**
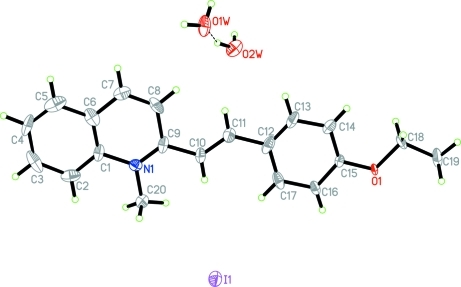
The asymmetric unit of the title compound. Displacement ellipsoids are drawn at the 50% probability level.

**Fig. 2.**
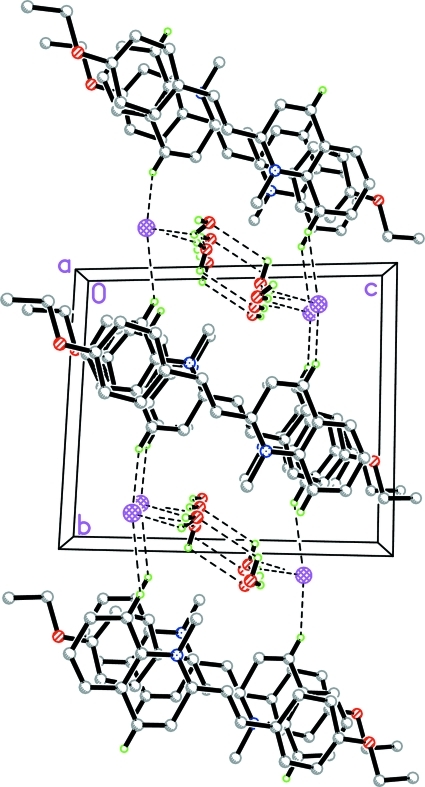
The crystal packing of the title compound, viewed down the a axis. H atom not involved in hydrogen bonding (dashed lines) have been omitted for clarity.

## supplementary crystallographic information

### Comment

Organic crystals are highly recognized as materials of the future because their molecular nature combined with versatility of synthetic chemistry can be used to alter their structures in order to maximize the nonlinear optical (NLO) properties (Kagawa *et al.*, 1994). The title quinolinium salt, (I), was synthesized in order to study its NLO properties. In addition, quinolinium derivatives were found to exhibit interesting bioactivities and pharmacological activities (Hopkins *et al.*, 2005; Kaminsky & Meltzer, 1968; Musiol *et al.*, 2006; O'Donnell *et al.*, 2010). Due to the well-known bioactivities of quinoline core, the antibacterial activities of (I) were also evaluated. Our results show that (I) is very active against the Methicillin-Resistant *Staphylococcus aureus* with a very low MIC value of 2.34 µg/ml, whereas it is inactive against the Gram-negative bacteria *i.e*. *Pseudomonas aeruginosa*, *Salmonella typhi* and *Shigella sonnei*. Nevertheless (I) did not possess NLO properties since it crystallized in the centrosymmetric triclinic P-*1* space group (Williams, 1984).

In the title compound (Fig. 1), the cation exists in an *E* configuration with respect to the ethenyl bond [torsion angle C9—C10—C11—C12 = -178.2 (8)°]. The cation is almost planar with a dihedral angle between the N1/C1–C9 quinolinium and C12–C17 benzene rings of 0.7 (4)°. The ethoxy unit is coplanar with the attached benzene ring with a C15—O1—C18—C19 torsion angle of 179.9 (6)°. Bond distances in the cation have normal values (Allen *et al.*, 1987) and are comparable to those observed in a related structure (Laksana *et al.*, 2008).

In the crystal, the cations are arranged into layers parallel to the (100) and stacked in anti-parallel manner along the *a* axis with π–π interactions involving the quinolinium ring system and benzene ring [Cg1···Cg2^ii^ = 3.678 (5) Å; symmetry code as in Table 1; Cg1 and Cg2 are centroids of the N1/C1/C6–C9 and C12–C17 rings, respectively]. The I^-^ ions and water molecules are located in the interstitial sites of the cations. The cations, I^-^ anions and water molecules are linked together through O—H···O, O—H···I and C—H···I hydrogen bonds (Table 1) into a two-dimensional network parallel to the (001) (Fig. 2).

### Experimental

The title compound was prepared by mixing a solution (1:1:1 mole ratio) of 1,2-dimethylquinolinium iodide (2.00 g, 7.0 mmol), 4-ethoxybenzaldehyde (4.32 ml, 7.0 mmol) and piperidine (0.69 ml, 7.0 mmol) in hot methanol (50 ml). The resulting solution was refluxed for 6 h under nitrogen atmosphere. The resultant orange-brown solid was filtered, washed with diethyl ether, dried in vacuo and purified by recrystallization. Brown needle-shaped single crystals of the title compound suitable for X-ray structure determination were obtained from methanol solution by slow evaporation at room temperature after several days (m.p. 492-494 K).

### Refinement

The water H atoms were initially located in a difference map and were refined with O–H and H···H distance restraints of 0.84 (1) and 1.37 (2) Å, respectively. During the final stages of the refinement they were allowed to ride on their parent O atoms with *U*_iso_(H) = 1.5*U*_eq_(O). The remaining H atoms were positioned geometrically and allowed to ride on their parent atoms, with C–H = 0.93 Å (aromatic) and 0.96 Å (CH_3_). The *U*_iso_ values were constrained to be 1.5*U*_eq_ of the carrier atom for methyl H atoms and 1.2*U*_eq_ for the remaining H atoms. The *U*_ij_ components of atoms C1, C6 and C9 were restrained to approximate isotropic behaviour. A rigid bond restraint with an s.u. of 0.01 was applied to the atomic displacement parameters of atoms C2 and C3 (also C12 and C17), because the components of the displacement parameters in the direction of the bond between these atoms were slightly inconsistent. A rotating group model was used for the methyl groups. The highest residual electron density peak is located at 1.17 Å from I1 and the deepest hole is located at 1.46 Å from C11.

### Crystal data

**Table:**

C_20_H_20_NO^+^·I^−^·2H_2_O	*Z* = 2
*M**_r_* = 453.30	*F*(000) = 456
Triclinic, *P*1	*D*_x_ = 1.566 Mg m^−^^3^
Hall symbol: -P 1	Melting point = 492–494 K
*a* = 8.2450 (9) Å	Mo *K*α radiation, λ = 0.71073 Å
*b* = 10.6676 (12) Å	Cell parameters from 3910 reflections
*c* = 12.2492 (14) Å	θ = 1.8–26.5°
α = 85.789 (2)°	µ = 1.68 mm^−^^1^
β = 70.516 (2)°	*T* = 100 K
γ = 71.272 (2)°	Needle, brown
*V* = 961.21 (19) Å^3^	0.49 × 0.08 × 0.05 mm

### Data collection

**Table:**

Bruker APEX DUO CCD area-detector diffractometer	3910 independent reflections
Radiation source: sealed tube	3551 reflections with *I* > 2σ(*I*)
graphite	*R*_int_ = 0.033
φ and ω scans	θ_max_ = 26.5°, θ_min_ = 1.8°
Absorption correction: multi-scan (*SADABS*; Bruker, 2009)	*h* = −10→10
*T*_min_ = 0.493, *T*_max_ = 0.927	*k* = −13→12
11172 measured reflections	*l* = −15→15

### Refinement

**Table:**

Refinement on *F*^2^	Primary atom site location: structure-invariant direct methods
Least-squares matrix: full	Secondary atom site location: difference Fourier map
*R*[*F*^2^ > 2σ(*F*^2^)] = 0.074	Hydrogen site location: inferred from neighbouring sites
*wR*(*F*^2^) = 0.211	H-atom parameters constrained
*S* = 1.15	*w* = 1/[σ^2^(*F*_o_^2^) + (0.1163*P*)^2^ + 8.5465*P*] where *P* = (*F*_o_^2^ + 2*F*_c_^2^)/3
3910 reflections	(Δ/σ)_max_ = 0.001
220 parameters	Δρ_max_ = 2.43 e Å^−^^3^
20 restraints	Δρ_min_ = −0.85 e Å^−^^3^

### Special details

**Table:**

Experimental. The crystal was placed in the cold stream of an Oxford Cryosystems Cobra open-flow nitrogen cryostat (Cosier & Glazer, 1986) operating at 100.0 (1) K.
Geometry. All esds (except the esd in the dihedral angle between two l.s. planes) are estimated using the full covariance matrix. The cell esds are taken into account individually in the estimation of esds in distances, angles and torsion angles; correlations between esds in cell parameters are only used when they are defined by crystal symmetry. An approximate (isotropic) treatment of cell esds is used for estimating esds involving l.s. planes.
Refinement. Refinement of F^2^ against ALL reflections. The weighted R-factor wR and goodness of fit S are based on F^2^, conventional R-factors R are based on F, with F set to zero for negative F^2^. The threshold expression of F^2^ > 2sigma(F^2^) is used only for calculating R-factors(gt) etc. and is not relevant to the choice of reflections for refinement. R-factors based on F^2^ are statistically about twice as large as those based on F, and R- factors based on ALL data will be even larger.

### Fractional atomic coordinates and isotropic or equivalent isotropic displacement parameters (Å^2^)

**Table:**

	*x*	*y*	*z*	*U*_iso_*/*U*_eq_	
I1	0.20275 (7)	0.86454 (5)	0.22772 (4)	0.0286 (2)	
O1	1.0063 (6)	0.2957 (5)	0.0164 (4)	0.0164 (10)	
N1	0.0190 (9)	0.6541 (6)	0.6219 (5)	0.0208 (13)	
C1	−0.1326 (9)	0.6672 (8)	0.7248 (6)	0.0217 (11)	
C2	−0.2476 (13)	0.7907 (9)	0.7709 (8)	0.0326 (19)	
H2A	−0.2303	0.8675	0.7351	0.039*	
C3	−0.3924 (12)	0.7963 (10)	0.8742 (9)	0.040 (2)	
H3A	−0.4731	0.8783	0.9064	0.048*	
C4	−0.4171 (11)	0.6817 (11)	0.9290 (7)	0.034 (2)	
H4A	−0.5106	0.6869	0.9985	0.041*	
C5	−0.3070 (14)	0.5671 (11)	0.8815 (8)	0.040 (2)	
H5A	−0.3259	0.4904	0.9167	0.048*	
C6	−0.1606 (11)	0.5559 (9)	0.7787 (8)	0.0286 (18)	
C7	−0.0408 (12)	0.4278 (9)	0.7286 (8)	0.0314 (18)	
H7A	−0.0604	0.3517	0.7646	0.038*	
C8	0.0966 (11)	0.4172 (8)	0.6319 (8)	0.0317 (19)	
H8A	0.1738	0.3342	0.6002	0.038*	
C9	0.1275 (9)	0.5398 (8)	0.5737 (6)	0.0217 (11)	
C10	0.2787 (10)	0.5185 (8)	0.4684 (6)	0.0237 (15)	
H10A	0.2917	0.5939	0.4280	0.028*	
C11	0.4004 (11)	0.4063 (9)	0.4220 (7)	0.0298 (17)	
H11A	0.3834	0.3316	0.4622	0.036*	
C12	0.5632 (11)	0.3792 (10)	0.3141 (7)	0.0322 (19)	
C13	0.6568 (11)	0.2555 (10)	0.2820 (7)	0.0325 (19)	
H13A	0.6225	0.1887	0.3276	0.039*	
C14	0.8067 (11)	0.2211 (9)	0.1813 (7)	0.0267 (16)	
H14A	0.8703	0.1327	0.1590	0.032*	
C15	0.8592 (9)	0.3215 (7)	0.1150 (6)	0.0155 (13)	
C16	0.7674 (10)	0.4541 (8)	0.1450 (6)	0.0225 (15)	
H16A	0.8050	0.5203	0.1003	0.027*	
C17	0.6132 (11)	0.4851 (9)	0.2471 (7)	0.0308 (18)	
H17A	0.5457	0.5728	0.2702	0.037*	
C18	1.0977 (11)	0.1578 (8)	−0.0216 (7)	0.0251 (16)	
H18A	1.0150	0.1195	−0.0369	0.030*	
H18B	1.1423	0.1076	0.0375	0.030*	
C19	1.2542 (11)	0.1552 (10)	−0.1316 (7)	0.037 (2)	
H19A	1.3064	0.0676	−0.1668	0.055*	
H19B	1.3445	0.1797	−0.1130	0.055*	
H19C	1.2106	0.2166	−0.1845	0.055*	
C20	0.0510 (12)	0.7731 (9)	0.5679 (8)	0.0340 (19)	
H20A	0.1758	0.7529	0.5203	0.051*	
H20B	0.0235	0.8385	0.6267	0.051*	
H20C	−0.0252	0.8067	0.5209	0.051*	
O1W	0.2568 (8)	0.0771 (7)	0.5862 (6)	0.0385 (15)	
H1W1	0.3030	−0.0003	0.6046	0.058*	
H2W1	0.1437	0.0966	0.6162	0.058*	
O2W	0.5594 (9)	0.1337 (7)	0.5759 (5)	0.0335 (14)	
H1W2	0.4515	0.1362	0.6067	0.050*	
H2W2	0.6181	0.0959	0.6196	0.050*	

### Atomic displacement parameters (Å^2^)

**Table:**

	*U*^11^	*U*^22^	*U*^33^	*U*^12^	*U*^13^	*U*^23^
I1	0.0254 (3)	0.0302 (3)	0.0278 (3)	−0.0099 (2)	−0.0040 (2)	−0.0019 (2)
O1	0.014 (2)	0.013 (2)	0.014 (2)	0.0002 (18)	0.0023 (18)	−0.0053 (18)
N1	0.025 (3)	0.021 (3)	0.018 (3)	−0.006 (2)	−0.011 (2)	0.001 (2)
C1	0.015 (2)	0.042 (3)	0.011 (2)	−0.010 (2)	−0.0062 (18)	−0.005 (2)
C2	0.042 (5)	0.033 (5)	0.041 (5)	−0.021 (4)	−0.029 (4)	0.016 (4)
C3	0.029 (4)	0.039 (5)	0.052 (5)	0.009 (4)	−0.026 (4)	−0.027 (4)
C4	0.021 (4)	0.071 (7)	0.014 (4)	−0.022 (4)	−0.001 (3)	−0.004 (4)
C5	0.055 (6)	0.054 (6)	0.035 (5)	−0.035 (5)	−0.032 (5)	0.020 (5)
C6	0.023 (3)	0.035 (4)	0.036 (4)	−0.003 (3)	−0.023 (3)	−0.010 (3)
C7	0.035 (4)	0.034 (5)	0.033 (5)	−0.017 (4)	−0.016 (4)	0.011 (4)
C8	0.025 (4)	0.019 (4)	0.048 (5)	−0.001 (3)	−0.012 (4)	−0.012 (4)
C9	0.015 (2)	0.042 (3)	0.011 (2)	−0.010 (2)	−0.0062 (18)	−0.005 (2)
C10	0.018 (3)	0.032 (4)	0.018 (3)	−0.004 (3)	−0.003 (3)	−0.007 (3)
C11	0.028 (4)	0.033 (5)	0.024 (4)	−0.008 (3)	−0.004 (3)	0.000 (3)
C12	0.026 (4)	0.058 (6)	0.013 (3)	−0.013 (4)	−0.008 (3)	0.004 (3)
C13	0.024 (4)	0.049 (6)	0.019 (4)	−0.014 (4)	0.001 (3)	0.005 (4)
C14	0.022 (4)	0.038 (5)	0.019 (4)	−0.010 (3)	−0.006 (3)	0.007 (3)
C15	0.010 (3)	0.023 (4)	0.014 (3)	−0.006 (3)	−0.004 (2)	−0.002 (3)
C16	0.018 (3)	0.025 (4)	0.019 (4)	0.005 (3)	−0.009 (3)	−0.007 (3)
C17	0.027 (4)	0.035 (5)	0.026 (4)	0.006 (3)	−0.014 (3)	−0.016 (3)
C18	0.027 (4)	0.017 (4)	0.024 (4)	0.006 (3)	−0.010 (3)	−0.009 (3)
C19	0.025 (4)	0.047 (6)	0.024 (4)	0.008 (4)	−0.006 (3)	−0.017 (4)
C20	0.031 (4)	0.034 (5)	0.026 (4)	−0.005 (3)	−0.003 (3)	0.007 (4)
O1W	0.016 (3)	0.057 (4)	0.034 (3)	−0.007 (3)	0.000 (2)	−0.007 (3)
O2W	0.043 (3)	0.047 (4)	0.023 (3)	−0.026 (3)	−0.015 (3)	0.001 (3)

### Geometric parameters (Å, °)

**Table:**

O1—C15	1.365 (8)	C11—H11A	0.93
O1—C18	1.450 (9)	C12—C13	1.308 (14)
N1—C9	1.300 (10)	C12—C17	1.435 (14)
N1—C1	1.426 (9)	C13—C14	1.393 (11)
N1—C20	1.447 (11)	C13—H13A	0.93
C1—C6	1.364 (13)	C14—C15	1.389 (11)
C1—C2	1.380 (13)	C14—H14A	0.93
C2—C3	1.410 (14)	C15—C16	1.383 (11)
C2—H2A	0.93	C16—C17	1.421 (11)
C3—C4	1.391 (15)	C16—H16A	0.93
C3—H3A	0.93	C17—H17A	0.93
C4—C5	1.303 (15)	C18—C19	1.517 (12)
C4—H4A	0.93	C18—H18A	0.97
C5—C6	1.404 (14)	C18—H18B	0.97
C5—H5A	0.93	C19—H19A	0.96
C6—C7	1.440 (13)	C19—H19B	0.96
C7—C8	1.319 (13)	C19—H19C	0.96
C7—H7A	0.93	C20—H20A	0.96
C8—C9	1.496 (12)	C20—H20B	0.96
C8—H8A	0.93	C20—H20C	0.96
C9—C10	1.435 (10)	O1W—H1W1	0.84
C10—C11	1.307 (12)	O1W—H2W1	0.84
C10—H10A	0.93	O2W—H1W2	0.84
C11—C12	1.502 (11)	O2W—H2W2	0.84
			
C15—O1—C18	116.7 (6)	C13—C12—C11	117.9 (8)
C9—N1—C1	122.7 (7)	C17—C12—C11	121.3 (8)
C9—N1—C20	118.8 (7)	C12—C13—C14	121.8 (9)
C1—N1—C20	118.6 (7)	C12—C13—H13A	119.1
C6—C1—C2	120.2 (7)	C14—C13—H13A	119.1
C6—C1—N1	119.1 (7)	C15—C14—C13	118.7 (8)
C2—C1—N1	120.7 (8)	C15—C14—H14A	120.6
C1—C2—C3	117.6 (8)	C13—C14—H14A	120.6
C1—C2—H2A	121.2	O1—C15—C16	115.6 (6)
C3—C2—H2A	121.2	O1—C15—C14	122.1 (7)
C4—C3—C2	121.4 (8)	C16—C15—C14	122.3 (7)
C4—C3—H3A	119.3	C15—C16—C17	117.2 (8)
C2—C3—H3A	119.3	C15—C16—H16A	121.4
C5—C4—C3	119.0 (8)	C17—C16—H16A	121.4
C5—C4—H4A	120.5	C16—C17—C12	119.1 (8)
C3—C4—H4A	120.5	C16—C17—H17A	120.5
C4—C5—C6	122.0 (9)	C12—C17—H17A	120.5
C4—C5—H5A	119.0	O1—C18—C19	106.7 (7)
C6—C5—H5A	119.0	O1—C18—H18A	110.4
C1—C6—C5	119.9 (8)	C19—C18—H18A	110.4
C1—C6—C7	119.4 (8)	O1—C18—H18B	110.4
C5—C6—C7	120.7 (9)	C19—C18—H18B	110.4
C8—C7—C6	120.7 (8)	H18A—C18—H18B	108.6
C8—C7—H7A	119.7	C18—C19—H19A	109.5
C6—C7—H7A	119.7	C18—C19—H19B	109.5
C7—C8—C9	119.6 (8)	H19A—C19—H19B	109.5
C7—C8—H8A	120.2	C18—C19—H19C	109.5
C9—C8—H8A	120.2	H19A—C19—H19C	109.5
N1—C9—C10	126.0 (8)	H19B—C19—H19C	109.5
N1—C9—C8	118.4 (7)	N1—C20—H20A	109.5
C10—C9—C8	115.6 (7)	N1—C20—H20B	109.5
C11—C10—C9	128.1 (8)	H20A—C20—H20B	109.5
C11—C10—H10A	115.9	N1—C20—H20C	109.5
C9—C10—H10A	115.9	H20A—C20—H20C	109.5
C10—C11—C12	130.2 (9)	H20B—C20—H20C	109.5
C10—C11—H11A	114.9	H1W1—O1W—H2W1	107.7
C12—C11—H11A	114.9	H1W2—O2W—H2W2	109.1
C13—C12—C17	120.9 (8)		
			
C9—N1—C1—C6	3.5 (10)	C20—N1—C9—C8	178.3 (7)
C20—N1—C1—C6	−177.8 (7)	C7—C8—C9—N1	1.4 (11)
C9—N1—C1—C2	−177.9 (7)	C7—C8—C9—C10	−179.2 (7)
C20—N1—C1—C2	0.8 (10)	N1—C9—C10—C11	173.5 (8)
C6—C1—C2—C3	0.0 (11)	C8—C9—C10—C11	−5.8 (12)
N1—C1—C2—C3	−178.6 (6)	C9—C10—C11—C12	−178.2 (8)
C1—C2—C3—C4	1.1 (11)	C10—C11—C12—C13	−175.1 (9)
C2—C3—C4—C5	−2.2 (12)	C10—C11—C12—C17	3.9 (14)
C3—C4—C5—C6	2.2 (12)	C17—C12—C13—C14	−0.9 (13)
C2—C1—C6—C5	0.0 (10)	C11—C12—C13—C14	178.1 (8)
N1—C1—C6—C5	178.6 (6)	C12—C13—C14—C15	1.3 (12)
C2—C1—C6—C7	179.1 (7)	C18—O1—C15—C16	−175.9 (6)
N1—C1—C6—C7	−2.3 (10)	C18—O1—C15—C14	4.8 (9)
C4—C5—C6—C1	−1.2 (12)	C13—C14—C15—O1	178.9 (7)
C4—C5—C6—C7	179.7 (8)	C13—C14—C15—C16	−0.4 (11)
C1—C6—C7—C8	0.8 (11)	O1—C15—C16—C17	179.9 (6)
C5—C6—C7—C8	179.9 (8)	C14—C15—C16—C17	−0.8 (10)
C6—C7—C8—C9	−0.3 (12)	C15—C16—C17—C12	1.1 (10)
C1—N1—C9—C10	177.7 (6)	C13—C12—C17—C16	−0.3 (12)
C20—N1—C9—C10	−1.1 (11)	C11—C12—C17—C16	−179.3 (7)
C1—N1—C9—C8	−3.0 (10)	C15—O1—C18—C19	179.9 (6)

### Hydrogen-bond geometry (Å, °)

**Table:**

*D*—H···*A*	*D*—H	H···*A*	*D*···*A*	*D*—H···*A*
O2W—H1W2···O1W	0.83	1.99	2.711 (11)	143
O1W—H2W1···I1^i^	0.84	2.77	3.588 (7)	163
O2W—H2W2···I1^ii^	0.84	2.87	3.579 (7)	144
C2—H2A···I1^iii^	0.93	3.02	3.814 (10)	145
C7—H7A···I1^i^	0.93	2.89	3.708 (10)	148

Symmetry codes: (i) −*x*, −*y*+1, −*z*+1; (ii) −*x*+1, −*y*+1, −*z*+1; (iii) −*x*, −*y*+2, −*z*+1.
